# Recurrence Pattern and Prognostic Value of Major Pathological Response in Patients with Locally Advanced Oesophageal Squamous Cell Carcinoma Treated with Neoadjuvant Immunochemotherapy: A Multicentre Retrospective Study

**DOI:** 10.3390/cancers18182902

**Published:** 2026-09-08

**Authors:** Feng Wang, Xiangyang Yu, Hao Chen, Dongjie Yan, Lei Yang, Ran Yang, Jianfei Zhu, Zhentao Yu, Yi Han

**Affiliations:** 1 Department of Minimally Invasive Surgery, Beijing Chest Hospital, Capital Medical University, Beijing Tuberculosis and Thoracic Tumor Research Institute, Beijing 101149, China; docwangfeng@126.com (F.W.); chenhaodoc@gmail.com (H.C.); yandj@yeah.net (D.Y.); 13501121043@139.com (L.Y.); 2 Department of Thoracic Surgery, National Cancer Center/National Clinical Research Center for Cancer/Cancer Hospital & Shenzhen Hospital, Chinese Academy of Medical Sciences and Peking Union Medical College, Shenzhen 518117, China; yuxy1991@yeah.net; 3 Department of Thoracic Surgery, Anyang Tumor Hospital, The Affiliated Anyang Tumor Hospital of Henan University of Science and Technology, Anyang 455000, China; yang1263@126.com; 4 Department of Thoracic Surgery, Shaanxi Provincial People’s Hospital, Xi’an 710068, China; zhujianfei718@163.com

**Keywords:** oesophageal cancer, neoadjuvant therapy, major pathologic response (MPR), overall survival (OS), recurrence-free survival (RFS), recurrence pattern

## Abstract

**Background/Objectives**: Major pathological response (MPR) has been suggested as a potential surrogate marker for assessing the efficacy of neoadjuvant therapy. However, its prognostic value and association with postoperative recurrence patterns remain unclear in patients with locally advanced oesophageal squamous cell carcinoma (ESCC) treated with neoadjuvant immunochemotherapy. This multicentre study aimed to investigate the relationship between MPR status, survival outcomes, and recurrence characteristics in this population. **Methods**: A total of 305 patients with locally advanced ESCC who received neoadjuvant immunochemotherapy followed by surgical resection at four medical centres in China between December 2019 and April 2024 were retrospectively enrolled in this study. Prognostic factors for overall survival (OS) and recurrence-free survival (RFS) were analyzed using Cox proportional hazards regression models. Propensity score matching (PSM) was performed to balance baseline characteristics between patients with and without MPR, and survival outcomes and postoperative recurrence patterns were subsequently compared. **Results**: Among the 305 patients, 115 (37.7%) achieved MPR. Multivariate analysis identified MPR and the number of neoadjuvant treatment cycles as independent prognostic factors for both OS and RFS (*p* < 0.05). After PSM, patients with MPR showed significantly improved OS and RFS compared with those without (*p* < 0.05). Postoperative recurrence occurred less frequently in the MPR group than in the non-MPR group (13.9% vs. 42.1%, respectively; *p* < 0.05), with reductions in both locoregional and distant recurrence. **Conclusions**: In patients with locally advanced ESCC receiving neoadjuvant immunochemotherapy and surgery, achieving MPR is associated with improved survival outcomes and a lower risk of postoperative recurrence. MPR may serve as a useful prognostic indicator for this population.

## Simple Summary

Oesophageal squamous cell carcinoma is a common and aggressive cancer with a high risk of recurrence after surgery. New treatments combining immunotherapy and chemotherapy before surgery have improved outcomes for some patients, but it remains unclear how to identify those who are most likely to benefit. This study evaluated whether a strong response to preoperative treatment, reflected by major pathological response, could predict long-term outcomes and patterns of cancer recurrence. By analyzing patients from multiple medical centres, we found that patients who achieved this response had longer survival and fewer recurrences after surgery compared with those who did not. These findings suggest that evaluating treatment response may help doctors better predict prognosis and develop more individualized follow-up and treatment strategies.

## 1. Introduction

Oesophageal cancer is among the most common malignancies worldwide, with oesophageal squamous cell carcinoma (ESCC) representing the predominant histological subtype in East Asia [1,2,3]. Owing to the absence of specific early symptoms, most patients are diagnosed at the locally advanced stage, for which multimodal treatment strategies are needed to improve long-term outcomes [1].

Neoadjuvant therapy has become an essential component of treatment for locally advanced ESCC. Although neoadjuvant chemoradiotherapy has long been considered the standard approach based on landmark trials such as NEOCRTEC5010 and CROSS, the integration of immune checkpoint inhibitors into perioperative treatment has recently reshaped the therapeutic landscape [4,5]. Emerging evidence suggests that neoadjuvant immunochemotherapy can achieve favourable pathological responses with acceptable safety profiles while potentially avoiding the increased surgical complexity and perioperative complications associated with radiotherapy [6,7,8,9]. Consequently, this strategy is being increasingly adopted in clinical practice.

With the evolution of neoadjuvant treatment, appropriate pathological response criteria for outcome evaluation have become increasingly important. Major pathological response (MPR), defined as a residual viable tumour of ≤10% in resected specimens, has been proposed as a meaningful indicator of treatment efficacy, and the prognostic relevance of MPR has been demonstrated in several solid cancers [10,11,12,13,14,15]. Compared with pathological complete response (pCR), achieving MPR ensures limited residual tumour, and this factor may better characterize pathological responses following neoadjuvant therapy.

However, the prognostic value of MPR in patients with locally advanced ESCC treated with neoadjuvant immunochemotherapy remains insufficiently explored, and its association with postoperative recurrence patterns has not been systematically evaluated. Therefore, this multicentre retrospective study investigated the relationships of MPR with survival outcomes and recurrence characteristics in patients with locally advanced ESCC who received neoadjuvant immunochemotherapy.

## 2. Materials and Methods

### 2.1. Patient Selection

This multicentre retrospective study included patients with locally advanced ESCC who underwent surgical resection following neoadjuvant immunochemotherapy at four tertiary medical centres in China between December 2019 and April 2024. All eligible patients were identified from institutional databases at the participating centres. Because this retrospective study included all eligible patients during the study period, no a priori sample size calculation was performed.

Patients were considered eligible for study inclusion if they met the following criteria: (1) had a histological diagnosis of ESCC confirmed by pre-treatment endoscopic biopsy, (2) received at least one cycle of neoadjuvant immunochemotherapy before surgery, and (3) underwent preoperative evaluation and treatment planning based on a discussion among the multidisciplinary team (MDT) members. Patients were excluded if they (1) had died within 30 days after surgery, (2) did not achieve R0 resection based on postoperative pathological assessment, or (3) lacked information on tumour differentiation in the final pathological report.

**Figure 1:**
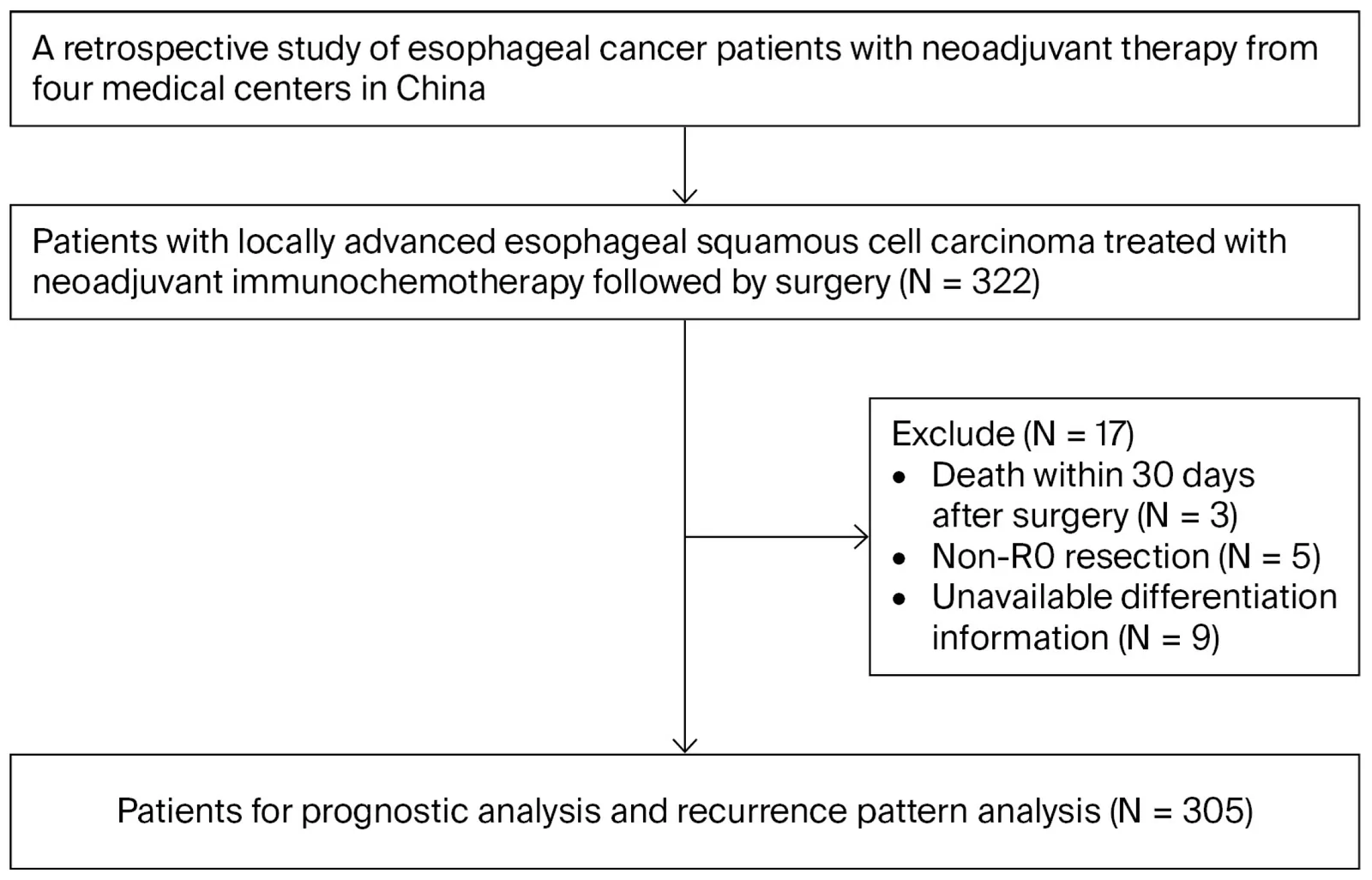
Flowchart of patient selection.

All biopsy and resection specimens were independently reviewed by two experienced pathologists at each participating centre who were blinded to survival and recurrence outcomes. The entire primary tumour bed was sampled and examined, and the percentage of residual viable tumour was visually estimated relative to the tumour bed. MPR was defined as ≤10% residual viable tumour in the primary tumour; lymph-node response was not included in the MPR classification. Disagreements were resolved by consultation with a senior pathologist and consensus review. Tumour staging was determined according to the 8th edition of the American Joint Committee on Cancer (AJCC) staging system for oesophageal cancer [16,17]. The patient selection process is summarized in Figure 1.

### 2.2. Treatment and Follow-Up

All patients were managed by an MDT including specialists in thoracic surgery, oncology, pathology, radiology, and radiotherapy. Decisions in terms of neoadjuvant regimens, surgical timing and approach, and postoperative management were made in accordance with current guidelines from the European Society for Medical Oncology (ESMO) and Chinese Society of Clinical Oncology (CSCO) [18,19].

Before neoadjuvant therapy, all patients underwent endoscopic examination for the pathological confirmation of oesophageal squamous cell carcinoma, along with baseline systemic evaluation. Preoperative assessments included computed tomography (CT) of the chest and abdomen, cranial magnetic resonance imaging (MRI), bone scintigraphy, and routine laboratory tests. Positron emission tomography–computed tomography (PET-CT) was performed selectively when clinically indicated. Immune checkpoint inhibitors were selected according to centre-specific practice and included sintilimab, pembrolizumab, nivolumab, tislelizumab, and camrelizumab. Chemotherapy consisted predominantly of a platinum agent plus nab-paclitaxel. One treatment cycle lasted three weeks.

After the completion of neoadjuvant immunochemotherapy, patients underwent surgical resection. Ivor Lewis or McKeown oesophagectomy with thoracic and abdominal lymph node dissection was routinely performed, and cervical lymphadenectomy was added when cervical lymph node involvement was suspected. The Sweet procedure was reserved for oesophagogastric junction tumours or for patients unsuitable for right-sided thoracotomy.

Patients underwent postoperative follow-up every three months during the first two years and annually thereafter. The routine follow-up included CT scans and hematological examinations, with additional investigations such as endoscopy or PET-CT performed when clinically indicated. Follow-up and time-to-event data were available for all 305 patients.

### 2.3. Data Analysis

Continuous variables are summarized using medians and ranges, and categorical variables were compared using appropriate contingency analyses. Survival endpoints were estimated using the Kaplan–Meier method and compared between the groups using the log-rank test. Variables demonstrating potential prognostic relevance in exploratory analyses were subsequently included in multivariable Cox regression models for identifying the independent predictors of outcomes. Variables with a *p* value of <0.10 in univariate analysis were included in multivariate analysis. The proportional hazards assumption was assessed using time-dependent covariates. Model discrimination was quantified using Harrell’s concordance index (C-index).

To minimize selection bias and any imbalance in baseline characteristics, propensity score matching (PSM) was performed using the 1:1 nearest-neighbour matching without replacement (calliper width = 0.05) and for clinically relevant covariates other than the response variable of interest. The propensity score model included age, sex, BMI, smoking history, alcohol consumption, family history, tumour differentiation, tumour length, clinical TNM stage, number of neoadjuvant treatment cycles, surgical procedure, and surgical approach. Covariate balance was assessed using standardized mean differences (SMD). Statistical significance was defined as a two-sided *p* value of <0.05. All the analyses were conducted using R statistical software and the optimal cut-off value was determined using X-tile software, version 3.6.1 (Yale University School of Medicine, New Haven, CT, USA).

## 3. Results

### 3.1. Baseline Characteristics

As summarized in Table 1, a total of 305 patients were included in the original cohort, of whom 115 achieved MPR and 190 were classified as non-MPR. The median age was 67 years; 66.9% of the patients were aged ≥63 years and 67.9% were male. Most patients had a body mass index (BMI) ranging between 18.5 and 23.9, suggesting that normal-weight individuals accounted for the majority of the study population.

Histopathologically, most tumours were moderately differentiated (54.1%), and most patients had stage III disease. A total of 84.3% of patients had tumours measuring ≥4 cm. Regarding treatment characteristics, 85.6% of the patients received one to two cycles of neoadjuvant immunochemotherapy and 14.4% received three or more. The McKeown procedure was the most frequently performed surgical approach (86.9%), followed by the Ivor Lewis and Sweet procedures. Minimally invasive surgery was performed in 71.1% of the patients.

As shown in Table 1, after 1:1 propensity score matching, 112 patients were included in the matched cohort, with 56 patients in each group. The absolute SMD before/after matching were 0.086/0.259 for age, 0.180/0.116 for sex, 0.175/0.000 for BMI, 0.036/0.179 for smoking history, 0.092/0.000 for alcohol consumption, 0.002/0.000 for family history, 1.312/0.087 for tumour differentiation, 0.034/0.000 for tumour length, 0.111/0.066 for clinical TNM stage, 0.016/0.000 for treatment cycles, 0.170/0.195 for surgical procedure, and 0.130/0.039 for surgical approach. Thus, matching markedly improved the major imbalance in tumour differentiation, although residual imbalance remained for age, sex, smoking history, and surgical procedure.

### 3.2. Survival Analysis

**Figure 2:**
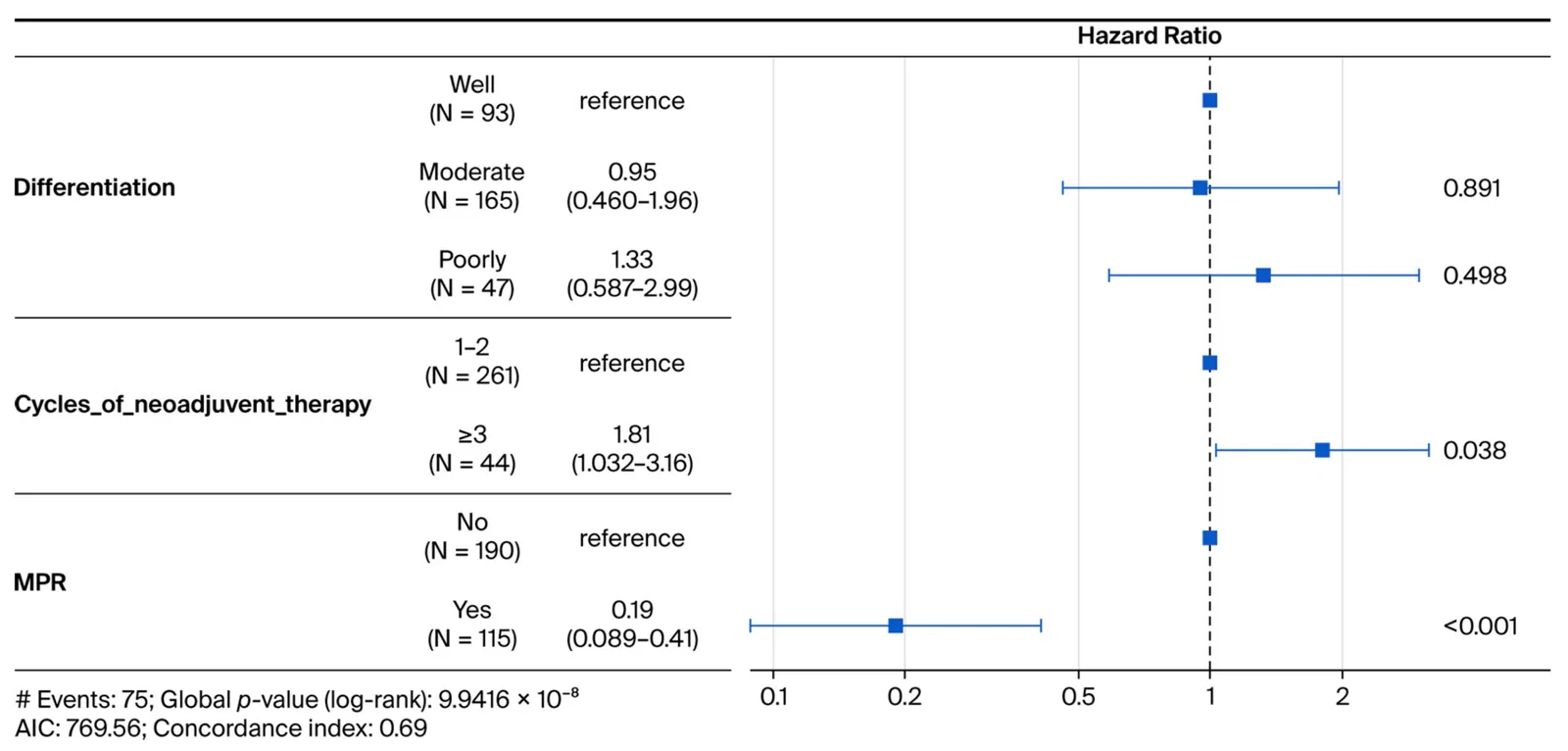
Forest plot of multivariate Cox proportional hazards regression analysis of overall survival (OS). Squares indicate hazard ratios (HRs), horizontal lines indicate 95% confidence intervals (CIs), and *p* values are shown for each covariate.

**Figure 3:**
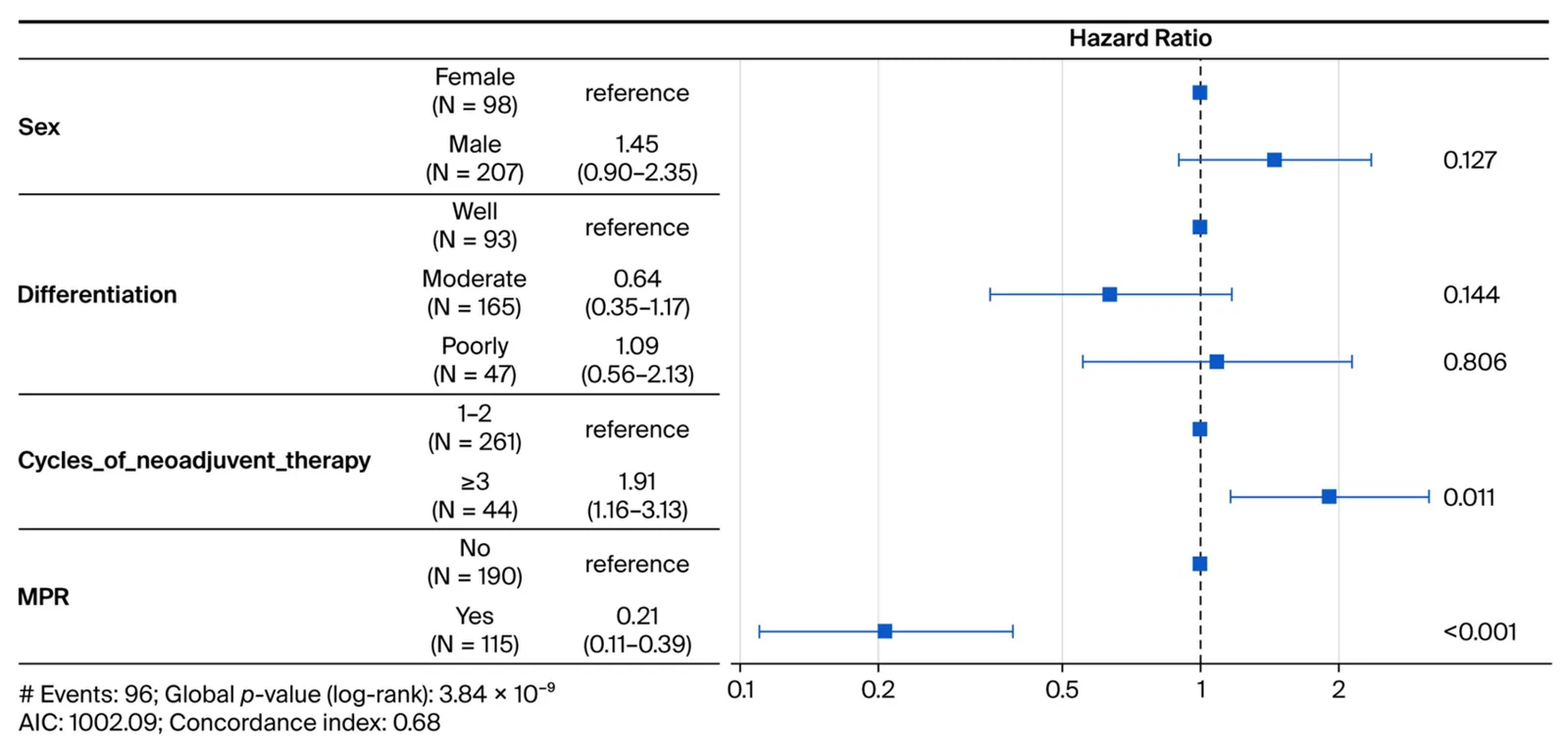
Forest plot of multivariate Cox proportional hazards regression analysis of recurrence-free survival (RFS). Squares indicate hazard ratios (HRs), horizontal lines indicate 95% confidence intervals (CIs), and *p* values are shown for each covariate.

Cox proportional hazards regression analysis was performed to identify the factors associated with survival outcomes. As illustrated in Table 2, in the univariate analysis, tumour differentiation, the number of neoadjuvant treatment cycles, and MPR were associated with OS. In addition, sex, tumour differentiation, the number of neoadjuvant treatment cycles, and MPR were associated with RFS. These variables were further evaluated in multivariate models. Multivariate analysis revealed that MPR (HR, 0.19; 95% CI, 0.09–0.41; *p* < 0.001) and three or more treatment cycles (HR, 1.81; 95% CI, 1.03–3.16; *p* = 0.038) were independently associated with OS (Figure 2). MPR (HR, 0.21; 95% CI, 0.11–0.39; *p* < 0.001) and three or more treatment cycles (HR, 1.90; 95% CI, 1.16–3.13; *p* = 0.011) were also independently associated with RFS (Figure 3). The proportional hazards assessment identified no evidence of violation for MPR or treatment cycles. The C-indices were 0.687 for the OS model and 0.682 for the RFS model.

**Figure 4:**
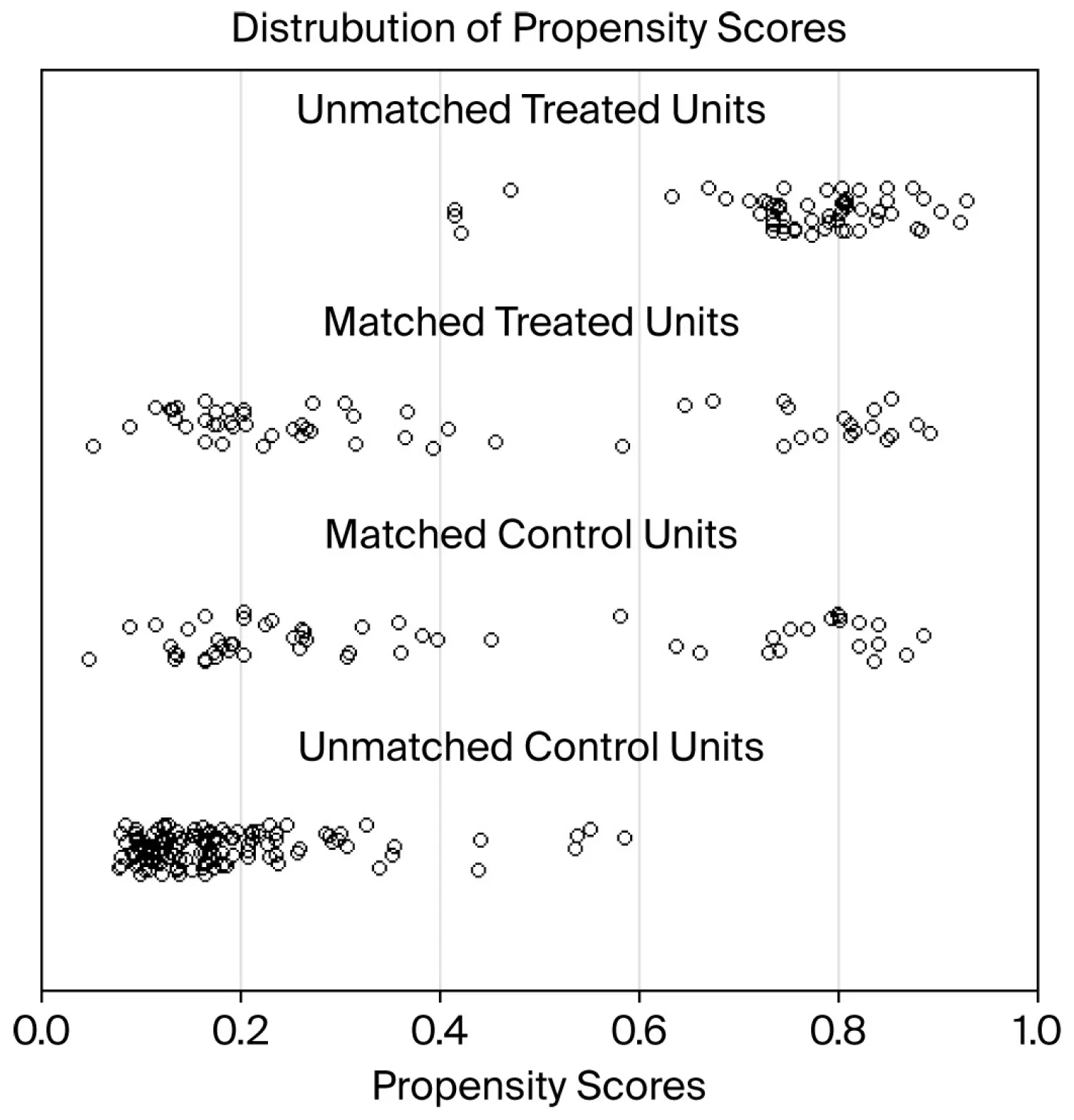
Distribution plot of the propensity scores before and after matching in the MPR and non-MPR groups. The improved overlap after matching indicates greater comparability between the groups, although some residual covariate imbalance remained.

**Figure 5:**
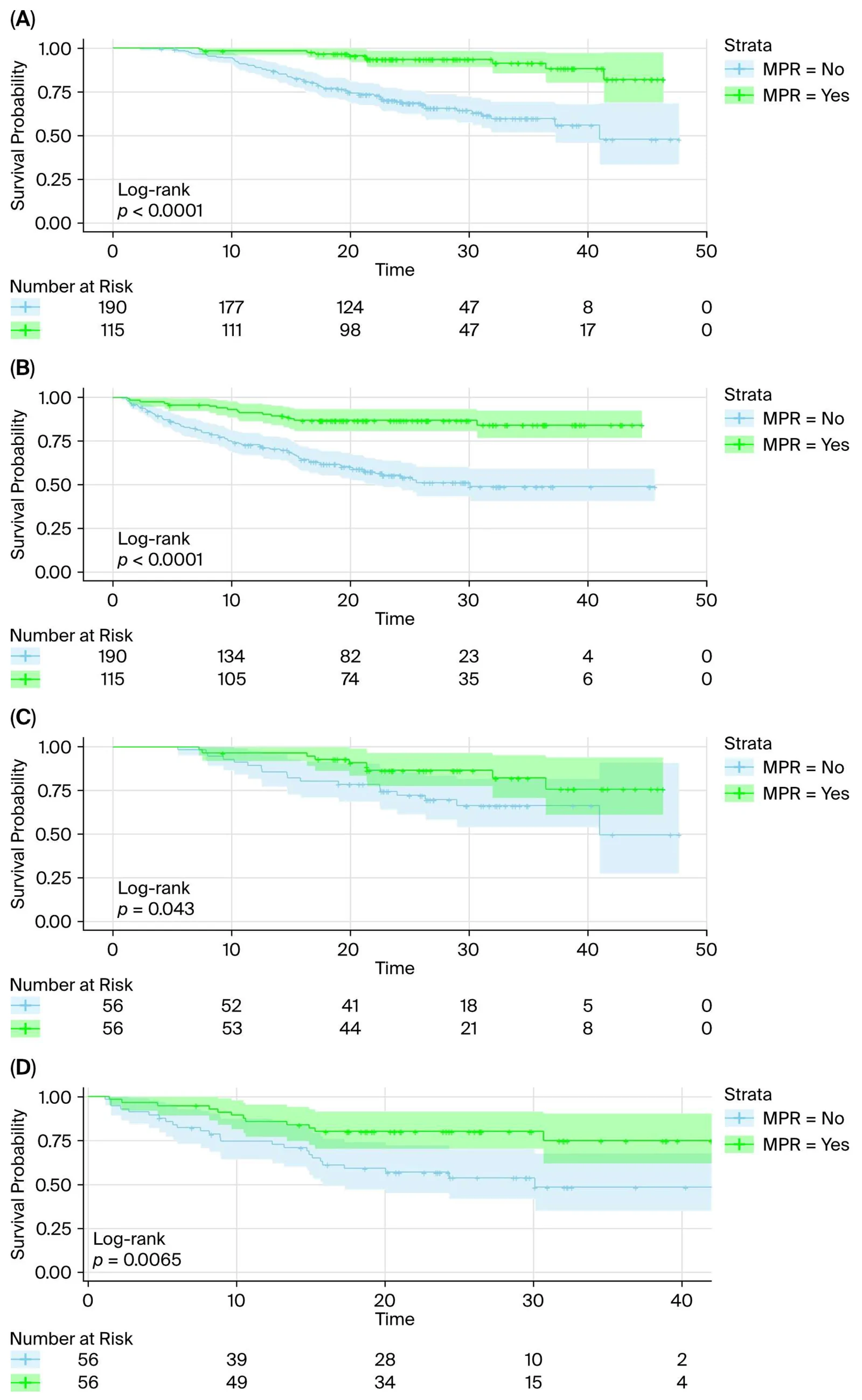
Kaplan–Meier survival curves with numbers at risk. Overall survival and recurrence-free survival (**A**,**B**) before and (**C**,**D**) after propensity score matching, respectively.

PSM was performed to minimize the impact of imbalances in baseline characteristics, resulting in a matched cohort of 112 patients, after which the distribution of propensity scores was more even between the MPR and non-MPR groups (Figure 4). Survival analyses revealed consistent results before and after PSM. As illustrated in Figure 5A,B, patients in the MPR group exhibited significantly improved OS and RFS compared with those in the non-MPR group in the original cohort. These differences remained significant in the matched cohort (Figure 5C,D), confirming the robustness of the association between MPR and survival outcomes (*p* < 0.05).

### 3.3. Recurrence Pattern

**Figure 6:**
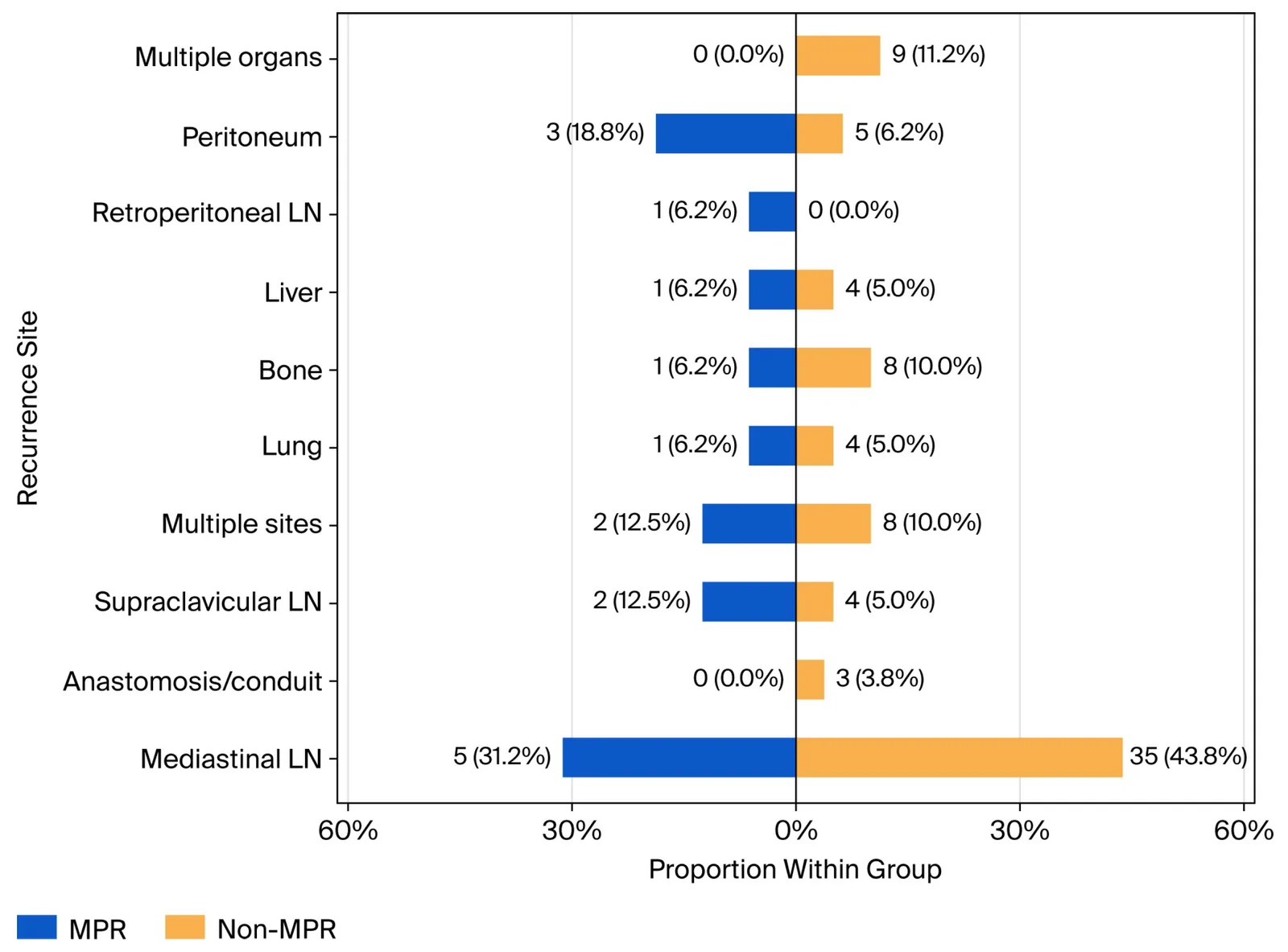
Butterfly plot showing the anatomical distribution of recurrence sites in the MPR (blue) and non-MPR (orange) groups. Percentages are calculated within each group; locoregional and distant recurrence comparisons are reported in Table 3.

The median follow-up duration, estimated using the reverse Kaplan–Meier method, was 27.0 months (observed range, 2.3–47.7 months). During follow-up, disease recurrence was observed in 96 of the 305 patients (31.5%). As shown in Table 3, the recurrence rate was significantly lower in the MPR group than in the non-MPR group (13.9% vs. 42.1%, *p* < 0.05). When recurrence events were stratified by anatomical distribution, both locoregional and distant recurrence were frequently detected in patients without MPR. Locoregional recurrence occurred in 7.8% and 26.3% of patients in the MPR and non-MPR groups, respectively (*p* < 0.001), whereas distant recurrence occurred in 6.1% and 15.8%, respectively (*p* = 0.011). The detailed distribution of recurrence sites is shown in Figure 6. In both groups, mediastinal lymph nodes were the most common site of recurrence. Notably, anastomotic recurrence and multiple-organ metastases were observed only in the non-MPR group. By contrast, no cases of multiple organ metastasis were recorded among patients in the MPR group. Among the distant metastatic sites, peritoneal involvement accounted for the highest proportion of patients in the MPR cohort. Distant recurrence was more widely distributed in the non-MPR group, with multiple-organ metastasis being the most frequent pattern.

## 4. Discussion

MPR is being increasingly recognized as an indicator of the efficacy of neoadjuvant therapy [10,20]. In the present multicentre cohort of patients with locally advanced ESCC treated with neoadjuvant immunochemotherapy, MPR was independently associated with improved OS and RFS, and this association persisted after adjustment for potential confounders using PSM. Our findings support MPR as a clinically relevant prognostic marker in this population.

The association between MPR and survival may be explained from both biological and clinical perspectives. Biologically, achieving MPR indicates the marked eradication of viable tumour cells within the primary lesion and regional lymphatic basin [10,21]. In the context of immunochemotherapy, this degree of tumour regression may reflect the effective activation of systemic antitumour immunity, which may extend beyond the resected specimen and target micrometastatic disease [14,22]. This proposed mechanism may reduce the risk of occult dissemination and later relapse, thereby contributing to improved RFS and OS [23,24]; however, the present study did not directly evaluate immune mechanisms. Clinically, MPR is a clear and repeatable standard for identifying patients with meaningful tumour regression and differentiating them from those with a limited response. This marker may distinguish patients with different risks, even when they are at similar clinical stages [10,20,21].

The prognostic value of MPR may be important in immune-based therapy. Immune checkpoint inhibitors can lead to active interactions between tumour cells and the immune system, and rather than having the same pattern in all patients, the pathological response may range from partial regression to complete clearance [25,26,27]. Therefore, if the treatment response is examined only on the basis of the pathological complete response, some patients with small residual tumours but clear survival benefits may be overlooked. MPR, defined as a residual viable tumour of ≤10%, can be used to identify these patients and provides a practical cut-off value that reflects a good biological response without the need for complete tumour clearance [11,12]. In this context, compared with stricter endpoints based only on complete or incomplete pathological response, MPR may better reflect the response pattern to immunotherapy.

Evidence from previous studies supports the prognostic relevance of pathological response across treatment settings. In ESCC, a multicentre cohort that included neo-adjuvant chemotherapy and chemoimmunotherapy identified MPR as a prognostic marker [10]. In non-small-cell lung cancer, MPR is associated with longer survival in patients who underwent neoadjuvant therapy, and it has been suggested as an intermediate endpoint in clinical trials [12,15]. Similar findings have been reported in head and neck squamous cell carcinoma and pancreatic cancer [13,28]. However, studies specifically focusing on ESCC patients receiving immunochemotherapy remain limited. Through the analysis of a multicentre cohort, this study adds to the current evidence and shows that MPR remains a useful prognostic indicator in the current immunochemotherapy setting. The observed association between three or more cycles and worse outcomes should not be interpreted as a harmful causal effect of additional therapy. In this retrospective cohort, patients receiving more cycles may have had lower responses, greater disease burden, treatment-related toxicity, or delayed surgery; these factors may therefore explain this counterintuitive association.

In addition to demonstrating the association between MPR and survival outcomes, our study revealed different recurrence patterns according to pathological response status. Although mediastinal lymph nodes were the most common site of recurrence in both groups, patients without MPR clearly had higher rates of locoregional recurrence and distant metastasis, including multiple-organ involvement. Previous studies on recurrence patterns in oesophageal cancer have also reported that nodal recurrence and distant metastasis are common after neoadjuvant therapy [29,30]. In our cohort, no multiple-organ metastasis was observed in the MPR group. This result suggests that achieving MPR may be related to not only better local tumour regression but also better systemic disease control.

The biological reason for these differences in recurrence remains hypothetical, it may be related to the ability of immunochemotherapy to induce durable immune surveillance [31]. Effective immune activation during neoadjuvant treatment may aid in eliminating micrometastatic lesions and controlling residual tumour cells with metastatic potential [27,31]. By contrast, failure to achieve MPR suggests resistance to treatment or weak immune activation, which may allow early tumour metastasis [21]. Because immune activation was not measured directly, the observed recurrence patterns cannot distinguish these mechanisms from differences in underlying tumour burden.

These findings also have practical clinical value. Adding MPR to postoperative risk assessment may help guide the formulation of more individualized follow-up strategies. Patients who do not achieve MPR may require intensified follow-up or additional systemic therapy, whereas those who do may be suitable for a more tailored monitoring plan. In addition, because MPR is associated with survival outcomes, it may also be used as a potential surrogate endpoint in future trials of neoadjuvant immunochemotherapy, as suggested for other tumour types [10,11,12,13,15].

Several limitations should be noted. First, the retrospective design and absence of a non-immunotherapy control group preclude attribution of the observed associations specifically to immunotherapy and may have introduced selection and survivorship bias. The study was restricted to patients who completed neoadjuvant therapy and underwent R0 resection, limiting generalizability to all patients beginning neoadjuvant treatment. Second, the median follow-up of 27.0 months may be adequate to capture many early recurrences but is insufficient to establish long-term OS and late recurrence; longer prospective follow-up is required. Third, treatment regimens and surgical approaches varied among centres. Centre identifiers were not retained in the final pooled deidentified analysis dataset; consequently, a centre-level random-effects model or stratified sensitivity analysis could not be performed, and residual centre-level confounding cannot be excluded. Finally, circulating tumour DNA was not routinely collected across the participating centres and therefore could not be included. Prospective studies combining standardized longitudinal ctDNA measurements with pathological response may clarify whether molecular residual disease improves recurrence prediction beyond MPR [32]. Combining pathological response with molecular biomarkers may improve prognostic stratification in future studies.

## 5. Conclusions

MPR was significantly associated with better survival and lower recurrence in patients with locally advanced ESCC who received neoadjuvant immunochemotherapy. The different recurrence patterns between the MPR and non-MPR groups further indicate that MPR may serve as a useful marker for risk stratification. Prospective studies are needed to confirm these results and to clarify how MPR should be incorporated into clinical decision-making.

## Figures and Tables

**Table 1:** Comparison of clinicopathological characteristics between the original and matched dataset.

Variables	Original Dataset				Matched Dataset			
	Total	MPR		*p* Value	Total	MPR		*p* Value
	N = 305	No (N = 190)	Yes (N = 115)		N = 112	No (N = 56)	Yes (N = 56)	
Age:				0.544				0.244
≤63	101 (33.1%)	60 (31.6%)	41 (35.7%)		43 (38.4%)	25 (44.6%)	18 (32.1%)	
>63	204 (66.9%)	130 (68.4%)	74 (64.3%)		69 (61.6%)	31 (55.4%)	38 (67.9%)	
Sex:				0.160				0.683
Female	98 (32.1%)	55 (28.9%)	43 (37.4%)		35 (31.2%)	19 (33.9%)	16 (28.6%)	
Male	207 (67.9%)	135 (71.1%)	72 (62.6%)		77 (68.8%)	37 (66.1%)	40 (71.4%)	
BMI:				0.331				1.000
<18.5	26 (8.5%)	15 (7.9%)	11 (9.5%)		10 (8.9%)	5 (8.9%)	5 (8.9%)	
18.5–23.9	186 (61.0%)	122 (64.2%)	64 (55.7%)		54 (48.2%)	27 (48.2%)	27 (48.2%)	
≥24	93 (30.5%)	53 (27.9%)	40 (34.8%)		48 (42.9%)	24 (42.9%)	24 (42.9%)	
Smoking:				0.855				0.450
No	177 (58.0%)	109 (57.4%)	68 (59.1%)		57 (50.9%)	31 (55.4%)	26 (46.4%)	
Yes	128 (42.0%)	81 (42.6%)	47 (40.9%)		55 (49.1%)	25 (44.6%)	30 (53.6%)	
Alcohol:				0.517				1.000
No	204 (66.9%)	124 (65.3%)	80 (69.6%)		76 (67.9%)	38 (67.9%)	38 (67.9%)	
Yes	101 (33.1%)	66 (34.7%)	35 (30.4%)		36 (32.1%)	18 (32.1%)	18 (32.1%)	
Family history:				1.000				1.000
No	220 (72.1%)	137 (72.1%)	83 (72.2%)		86 (76.8%)	43 (76.8%)	43 (76.8%)	
Yes	85 (27.9%)	53 (27.9%)	32 (27.8%)		26 (23.2%)	13 (23.2%)	13 (23.2%)	
Differentiation:				<0.001				0.885
Well	93 (30.5%)	20 (10.5%)	73 (63.5%)		36 (32.1%)	18 (32.1%)	18 (32.1%)	
Moderate	165 (54.1%)	138 (72.6%)	27 (23.5%)		52 (46.4%)	27 (48.2%)	25 (44.6%)	
Poorly	47 (15.4%)	32 (16.8%)	15 (13.0%)		24 (21.4%)	11 (19.6%)	13 (23.2%)	
Length:				0.896				1.000
<4 cm	48 (15.7%)	29 (15.3%)	19 (16.5%)		8 (7.1%)	4 (7.1%)	4 (7.1%)	
≥4 cm	257 (84.3%)	161 (84.7%)	96 (83.5%)		104 (92.9%)	52 (92.9%)	52 (92.9%)	
cTNM:				0.437				1.000
II	38 (12.5%)	21 (11.1%)	17 (14.8%)		9 (8.0%)	4 (7.1%)	5 (8.9%)	
III	267 (87.5%)	169 (88.9%)	98 (85.2%)		103 (92.0%)	52 (92.9%)	51 (91.1%)	
Cycles of neoadjuvant therapy:				1.000				1.000
1–2	261 (85.6%)	163 (85.8%)	98 (85.2%)		98 (87.5%)	49 (87.5%)	49 (87.5%)	
≥3	44 (14.4%)	27 (14.2%)	17 (14.8%)		14 (12.5%)	7 (12.5%)	7 (12.5%)	
Surgery:				0.311				0.560
McKeown	265 (86.9%)	165 (86.9%)	100 (87.0%)		94 (83.9%)	49 (87.5%)	45 (80.4%)	
Ivor Lewis	29 (9.5%)	16 (8.4%)	13 (11.3%)		15 (13.4%)	6 (10.7%)	9 (16.1%)	
Sweet	11 (3.6%)	9 (4.7%)	2 (1.7%)		3 (2.7%)	1 (1.8%)	2 (3.5%)	
Approach:				0.337				1.000
Minimally	217 (71.1%)	131 (68.9%)	86 (74.8%)		79 (70.5%)	40 (71.4%)	39 (69.6%)	
Open	88 (28.9%)	59 (31.1%)	29 (25.2%)		33 (29.5%)	16 (28.6%)	17 (30.4%)	

**Table 2:** Univariate analysis of overall survival and recurrence-free survival before propensity score matching.

Variables	Total	Overall Survival		Recurrence-Free Survival	
		HR (95% CI)	*p* Value	HR (95% CI)	*p* Value
Age:			0.203		0.101
≤63	101 (33.1%)	Reference		Reference	
>63	204 (66.9%)	1.39 (0.84–2.30)		1.46 (0.93–2.28)	
Sex:			0.320		0.020
Female	98 (32.1%)	Reference		Reference	
Male	207 (67.9%)	1.29 (0.78–2.14)		1.75 (1.09–2.82)	
BMI:			0.932		0.103
<18.5	26 (8.5%)	Reference		Reference	
18.5–23.9	186 (61.0%)	1.02 (0.44–2.39)		1.29 (0.55–3.00)	
≥24	93 (30.5%)	0.93 (0.37–2.29)		1.92 (0.81–4.56)	
Smoking:			0.516		0.266
No	177 (58.0%)	Reference		Reference	
Yes	128 (42.0%)	1.16 (0.74–1.83)		1.26 (0.84–1.87)	
Alcohol:			0.206		0.205
No	204 (66.9%)	Reference		Reference	
Yes	101 (33.1%)	1.35 (0.85–2.14)		1.30 (0.86–1.96)	
Family history:			0.449		0.390
No	220 (72.1%)	Reference		Reference	
Yes	85 (27.9%)	1.21 (0.74–1.98)		1.21 (0.78–1.86)	
Differentiation:			0.004		0.004
Well	93 (30.5%)	Reference		Reference	
Moderate	165 (54.1%)	2.48 (1.31–4.69)		1.68 (1.01–2.82)	
Poorly	47 (15.4%)	3.17 (1.51–6.65)		2.68 (1.47–4.87)	
Length:			0.201		0.827
<4 cm	48 (15.7%)	Reference		Reference	
≥4 cm	257 (84.3%)	1.61 (0.77–3.34)		1.07 (0.60–1.88)	
cTNM:			0.776		0.468
II	38 (12.5%)	Reference		Reference	
III	267 (87.5%)	1.11 (0.55–2.23)		1.27 (0.66–2.45)	
Cycles of neoadjuvant therapy:			0.045		0.009
1–2	261 (85.6%)	Reference		Reference	
≥3	44 (14.4%)	1.75 (1.00–3.03)		1.88 (1.16–3.05)	
Surgery:			0.279		0.210
McKeown	265 (86.9%)	Reference		Reference	
Ivor Lewis	29 (9.5%)	1.66 (0.85–3.24)		0.87 (0.42–1.80)	
Sweet	11 (3.6%)	1.43 (0.52–3.95)		2.02 (0.88–4.62)	
Approach:			0.175		0.217
Minimally	217 (71.1%)	Reference		Reference	
Open	88 (28.9%)	1.38 (0.86–2.21)		1.31 (0.85–2.00)	
MPR:			<0.001		<0.001
No	190 (62.3%)	Reference		Reference	
Yes	115 (37.7%)	0.19 (0.10–0.38)		0.25 (0.15–0.43)	

**Table 3:** Comparison of recurrence patterns between the MPR and non-MPR groups.

Variables	Total (N = 305)	non-MPR (N = 190)	MPR (N = 115)	*p* Value
Recurrence:				<0.001
No	209 (68.5%)	110 (57.9%)	99 (86.1%)	
Yes	96 (31.5%)	80 (42.1%)	16 (13.9%)	
Recurrence site:				<0.001
No	209 (68.5%)	110 (57.9%)	99 (86.1%)	
Local	59 (19.3%)	50 (26.3%)	9 (7.8%)	
Distant	37 (12.1%)	30 (15.8%)	7 (6.1%)	

## Data Availability

The data of this study are available from the corresponding authors upon reasonable request.

## Abbreviations

ESCC	oesophageal squamous cell carcinoma
MPR	major pathological response
PSM	propensity score matching
OS	overall survival
RFS	recurrence-free survival
ESMO	European Society for Medical Oncology
CSCO	Chinese Society of Clinical Oncology
CT	computed tomography
MRI	magnetic resonance imaging
PET-CT	positron emission tomography–computed tomography
